# DNA Methylation in Algae and Its Impact on Abiotic Stress Responses

**DOI:** 10.3390/plants12020241

**Published:** 2023-01-05

**Authors:** Michele Ferrari, Antonella Muto, Leonardo Bruno, Radiana Cozza

**Affiliations:** Department of Biology, Ecology and Earth Science, University of Calabria, Ponte P. Bucci, Arcavacata di Rende, 87036 Cosenza, Italy

**Keywords:** abiotic stress responses, algae, DNA methylation, heavy metal, light, nutrient, temperature

## Abstract

Epigenetics, referring to heritable gene regulatory information that is independent of changes in DNA sequences, is an important mechanism involved both in organism development and in the response to environmental events. About the epigenetic marks, DNA methylation is one of the most conserved mechanisms, playing a pivotal role in organism response to several biotic and abiotic stressors. Indeed, stress can induce changes in gene expression through hypo- or hyper-methylation of DNA at specific loci and/or in DNA methylation at the genome-wide level, which has an adaptive significance and can direct genome evolution. Exploring DNA methylation in responses to abiotic stress could have important implications for improving stress tolerance in algae. This article summarises the DNA methylation pattern in algae and its impact on abiotic stress, such as heavy metals, nutrients and temperature. Our discussion provides information for further research in algae for a better comprehension of the epigenetic response under abiotic stress, which could favour important implications to sustain algae growth under abiotic stress conditions, often related to high biosynthesis of interesting metabolites.

## 1. Introduction

Algae, as well as plants, are continuously exposed to a variety of biotic and abiotic stresses, which could potentially reduce their growth, development, productivity and survival [1]. Tolerance, avoidance, and resistance are three major strategies followed by plants to counter the recurring biotic and abiotic stresses [2]. These mechanisms are very complex and involve the activation of many genes and the interconnection among various signalling pathways regulating numerous metabolic networks.

The acclimation of plants to adverse environmental conditions requires reversible, as well as persistent, epigenetic modulation of gene expression.

The modifications of DNA methylation patterns allow rapid and reversible changes in the chromatin structure, enabling the activation of defence pathways through the combination of genetic and epigenetic mechanisms [3]. DNA methylation, together with other epigenetic marks, such as the histone code (post-translational modifications in histone proteins) and non-coding RNAs (ncRNAs), contributes to establishing the “epigenome” that, unlike the stable genome, is dynamically altered during both developmental processes and in response to different environmental factors and/or stresses [4,5,6,7,8,9]. So, the single genome in a plant cell could give rise to multiple epigenomic changes in response to developmental and environmental cues.

DNA methylation has been reported to play an important role in plant adaptive responses to environmental stresses [10,11,12]. Stresses can induce changes in gene expression through hypomethylation or hypermethylation of DNA-specific loci [13,14], usually associated with transcriptional regulation of genes involved in plant stress responses [14,15,16]. These genes, in turn, control important genetic functions, such as transcription, replication, DNA repair, gene transposition and cell differentiation [17,18,19]. DNA methylation may also involve genome regions, which have an adaptive significance during stress responses and can direct genome evolution [20].

Persistent stress is vital for establishing DNA methylation-dependent stress memory in plants [21]. On the other hand, stress-induced epigenetic modifications are not only persistent during exposure to stress but can also be transmitted hereditarily, thus ensuring the transfer of “stress memory” to progeny [22]. If the progeny is not continuously stressed, the inherited epigenetic status is gradually reset [23]. In fact, in the presence of persistent stress, the epigenetic mechanism establishes a DNA methylation-dependent stress memory in plants, mainly due to GC-rich sequences methylation [21,23,24]; this mechanism ensures the faithful transferring of the “stress memory” to the offspring [25]. Despite changes in DNA methylation associated with environmental stress have been reported [26,27], epigenetic factors involved in the stress response and their implications in algae remain poorly understood [28,29]. The current understanding of DNA methylation in algae and the recent progress in the epigenetic reprogramming involved in algal environmental responses will be discussed and summarised in this review.

## 2. DNA Methylation

DNA methylation is a conserved epigenetic modification crucial for various biological processes, including gene and transposon silencing, imprinting and X chromosome inactivation. Together with other epigenetic marks, such as the histone code, post-translational modifications and small RNA interference, DNA methylation contributes to chromatin remodelling processes, and it is involved in the regulation of gene expression without altering the primary DNA sequence [4,5].

Although all nucleotides can be methylated, DNA methylation generally refers to an addition of a methyl group through a covalent modification onto the C5-cytosine to form 5-methylcytosine (5-mC) [30], as well as onto the N6-adenine to form 6-methyladenine (6mA) [31].

DNA methylation can be found both in prokaryotes and eukaryotes. In bacteria, 6mA is a prevalent form of DNA methylation that plays an important role in DNA repair and replication. Bacterial DNA methylation can also act as a defence mechanism against invading phage; in fact, methylation differentiates the host from the phage genome, which becomes the preferential target of the cleavage action of host restriction enzymes [13].

In eukaryotes, DNA methylation is a fundamental mechanism for the maintenance of genome stability and the regulation of gene expression in response to both external and internal stimuli, thus playing a relevant role in plant diversity and development [14,32,33,34]. In plants, DNA methylation is more extensive and affects a wider sequence diversity than in animal genomes. In animals, cytosine methylation is mainly restricted to the symmetric CpG dinucleotide, except for the embryonic stem cells [35], the adult mouse cortex and the human brain [36], where CpH methylation was found. By contrast, plants have relatively high concentrations of 5-mC compared to non-plant species because cytosine methylation occurs in three sequence contexts: symmetric CG and asymmetric CHG and CHH (where H = A, T or C) [37].

The main studies of mechanisms on DNA methylation derive from the model species *Arabidopsis thaliana.* The dynamic of DNA methylation is controlled by specific DNA methyltransferases that mediate cytosine methylation in the different sequence contexts [4,38].

Cytosine-5 DNA methyltransferases recognise a specific DNA sequence and catalyse the transfer of a methyl group from the cofactor *S*-adenosyl-l-methionine (AdoMet) to carbon 5 in the pyrimidine ring of cytosine residues [39]. Different C5-cytosine methyltransferases have been characterised in prokaryotes and eukaryotes. All methyltransferases share a catalytic domain containing 10 conserved small motifs (annotated I to X), suggesting a common origin [40].

**Figure 1:**
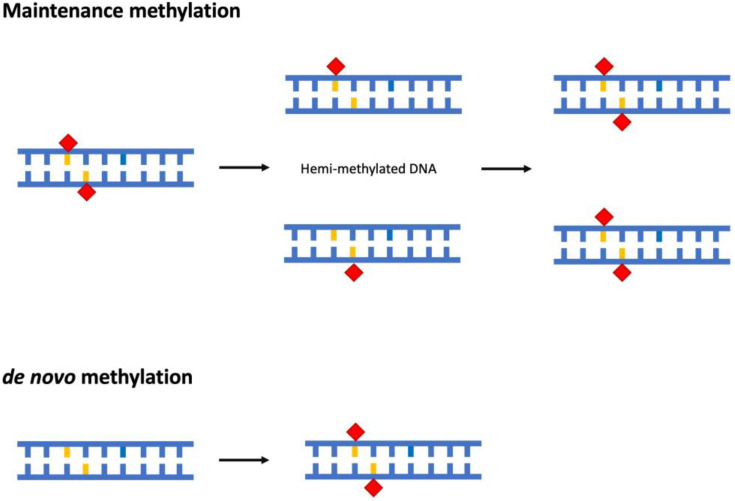
Schematic representation of maintenance and de novo methylation of DNA. The methyl group is indicated by red rhombus, whereas cytosine is indicated in orange.

DNA methylation can be distinguished in maintenance and de novo methylation. Maintenance methylation preserves the methylation status of symmetric (palindromic) sites after DNA replication through the recognition of hemi-methylated sites and methylation of the newly synthesised filament [41,42] (Figure 1). On the other hand, de novo methylation consists in the methylation of DNA sequences not previously methylated (Figure 1); in plants, an RNA-directed DNA methylation pathway is crucial for this process [43]. De novo methylation is involved in the rearrangement of methylation patterns during embryogenesis and in the cell differentiation processes during development [44].

In land plants, three DNA methyltransferase subfamilies have been implicated in the establishment and/or maintenance of DNA methylation [45,46], namely the DNA METHYLTRANSFERASEs (METs), CHROMOMETHYLASEs (CMTs) and DOMAINS-REARRANGED METHYLTRANSFERASEs (DRMs) subfamilies.

The plant homolog of mammalian DNA methyltransferase 1 (DNMT1), namely MET1, is the major CG maintenance methyltransferase. In plants DNMT2 homologs are also present that, like mammalian DNMT2, possess transfer RNA (tRNA) methylase activity [47]. Recent in vitro research has provided evidence that DNMT2 can efficiently methylate DNA when DNA fragments are presented as covalent DNA–RNA hybrids in the structural context of tRNAs [48].

The plant DRMs and their mammalian homologs, the DNMT3 group, are predominantly de novo methyltransferases. The domains of amino- and carboxy-terminal halves of DRM proteins are arranged in reverse order when compared with DNMT3 (domains VI–X are followed by I–V). DRM2 catalyses cytosine methylation in all sequence contexts and is the prominent cytosine methyltransferase in the RNA-directed DNA methylation pathway [49].

CMTs, identified exclusively in plants, are characterised by the presence of an amino acid chromodomain motif, between the conserved motifs II and IV, which binds to methylated histones. CMT3 is the enzyme primarily responsible for CHG maintenance methylation [38,46]. CMT2 plays a role in maintaining CHH methylation in specific genomic contexts, such as the central regions of large transposable elements, presumably via cross-talk with histone modifications like its paralog, CMT3 [50].

Methyl marks were also discovered on adenine bases in DNA (6 mA) [51]. The distribution and function of 6 mA in eukaryotes had remained largely unknown until several recent studies reported 6 mA distribution patterns in the genomes of the unicellular green algae *Chlamydomonas reinhardtii*, fungi and several multicellular animals [52]. In *C. reinhardtii*, 6 mA is mainly located at AT dinucleotides near transcription start sites and shares little correlation with 5-mC and seems to mark the transcriptional start sites of active genes [51]. More recently, also in the *A. thaliana* genome, 6 mA was found. The occurrence of 6 mA is more frequent in gene bodies than intergenic regions and positively correlates with the gene expression level and the transition from vegetative to reproductive growth in *A. thaliana* [52].

Plants have also evolved an active DNA demethylation mechanism that directly excises 5-mC and replaces it with unmethylated C via the base excision repair (BER) pathway by 5-mC DNA glycosylases [53,54]. Plant 5-mC DNA glycosylases are grouped in the DEMETER-LIKE (DML) family, which are unusually large DNA glycosylases. So far, they have only been detected in plants, including mosses (e.g., *Phycomitrella patens*) and unicellular green algae (e.g., *Ostreococcus*), suggesting that active demethylation through 5-mC excision arose early during plant evolution [53]. Active DNA demethylation serves important functions during various stages of the plant life cycle, including seed germination, fruit ripening and responses to a variety of stress conditions [55].

### 2.1. Maintenance of DNA Methylation

Maintenance of plant DNA methylation depends on the cytosine sequence context and it is catalysed by DNA methyltransferases, which, in turn, are regulated by different mechanisms. The maintenance of already established DNA methylation patterns occurs in all the different sequence contexts: CG, CHG and CHH. The maintenance of CG methylation in plants requires the activity of MET1, the VARIANT IN METHYLATION (VIM) and the DDM1 chromatin remodelling factor. VIMs are a family of SRA (SET and RING-associated) domain proteins orthologs of mammalian ubiquitin-like PHD and RING finger domains 1 (UHRF1) [4], suggesting that both plants and mammals maintain CG methylation in a similar manner. MET1 is associated with DNA replication sites, it recognizes hemi-methylated CG dinucleotides following DNA replication and methylates the unmodified cytosine in the daughter strand. MET1 has been proposed to be recruited to DNA by VIM proteins in a similar way to how DNMT1 is recruited by UHRF1 [14].

The maintenance of methylation at CHG sites does not seem to depend on the palindromic symmetry of the sequence and appears to involve histone methylation [56]. Genome-wide analyses showed that lysine 9 on histone 3 (H3K9) dimethylation and DNA methylation are highly correlated [57]. Indeed CMT3 is largely responsible for maintaining CHG methylation [58] and its activity is strongly associated with the dimethylation of H3K9, where the dual recognition of H3K9me2 by BAH (bromo adjacent homology) and CHROMO domains of CMT3 leads to the methylation of CHG sites [46].

In more detail, SUVH4, also known as histone H3K9 methyltransferase KRYPTONITE (KYP), has been demonstrated to be able to bind methylated CHH or CHG of the DNA through its SRA domain [59]. CMT3 is recruited by H3K9me and further methylates CHG sites of the DNA to create binding sites for KYP, resulting in a self-reinforcing feedback loop [4]. A loss of these two components results in a dramatic decrease in DNA methylation [59]. Two other H3K9 methyltransferases, SUVH5 and SUVH6, also contribute to global levels of CHG methylation [60]. Such interplay between DNA and histone methylation is also observed in mammals and, in many cases, the connection between these modifications appears to involve protein–protein interactions between the histone and DNA methyltransferases themselves. Whereas the direct protein interactions between CMT3 and KYP help to maintain CHG methylation in mammals, this mechanism has yet to be verified in plants.

Methylation in the CHH asymmetric context is maintained by DRM2 and RNA-directed DNA methylation (RdDM), which is involved in de novo methylation. However, at some loci, this kind of methylation is provided by CMT3 and DRM2 [61].

### 2.2. De Novo DNA Methylation

In plants, de novo DNA methylation is mediated through the RNA-directed DNA methylation (RdDM) pathway, which involves small interfering RNAs (siRNAs) and scaffold RNAs, in addition to an array of proteins. The mechanism for RdDM has been divided into the canonical RNA Polymerase (RNA Pol IV) and non-canonical (RNA Pol II) pathways. Both rely on the generation of siRNA molecules that direct the activity of DRM methyltransferases towards targets through sequence homology [43].

The canonical RdDM pathway relies on the activity of two plant-specific RNA Pol II paralogs, RNA Pol IV and RNA Pol V [62]. Canonical RdDM initiates with the recruitment of RNA Pol IV, preferentially towards heterochromatic regions by SAWADEE HOMEODOMAIN HOMOLOG 1 (SHH1) [63] and by RNA-DIRECTED RNA POLYMERASE 2 (RDR2) to produce double-stranded RNA (dsRNA) [64]. The putative chromatin remodelled CLASSY 1 (CLSY1) is also required for a correct RNA Pol IV and RDR2 recruitment and generation of the corresponding transcripts [65]. The dsRNA is processed by DICER-LIKE 3 (DCL3) into 24-nucleotide siRNAs that are subsequently methylated at their 3’ ends by HUA ENHANCER 1 (HEN1) and incorporated into ARGONAUTE 4 (AGO4). Pol V, in the nucleus, transcribes a scaffold RNA that base-pairs with AGO4-bound siRNAs. A key role is played by RNA-DIRECTED DNA METHYLATION 1 (RDM1), the only protein that interacts with both AGO4 and DRM2, thus creating a bridge between them [66]. Through this action, the canonical RdDM machinery acts to maintain methylation levels, predominantly in the CHH context, through de novo methylation at heterochromatic regions for continued silencing [65].

The canonical RdDM pathway utilises transcripts produced by RNA Pol II to de novo initiate new regions for silencing independently from RNA Pol IV [38]. RDR6 converts the RNA Pol II-mediated transcripts into dsRNA molecules that are subsequently processed by DCL2 and DCL4 to produce 21–22 nt siRNA [67,68]. These 21–22 nt siRNA can then be loaded into either AGO4 or AGO6 to direct methylation in the CHH context in an RNA Pol V- and DRM-dependent manner, as described above [69]. The non-canonical pathway could be considered to act as a surveillance mechanism aimed to identify and target actively transcribed regions for silencing, such as at active TE elements, potentially due to the loss of silencing factors or recent transposition [70].

In order to prevent aberrant hyper-methylation, the activity of RdDM is antagonised, and, therefore, moderate, by REPRESSOR OF SILENCING 1 (ROS1) [65]. ROS1, belonging to the DML family, is involved in the BER pathway and is crucial for the removal of methyl groups, preventing gene hypermethylation and the spreading of methylation from methylated TEs into genes [71].

### 2.3. DNA Methyltransferase in Algae

DNA methylation machinery contributes to the biological diversity of algae as they contain several DNA methyltransferases responsible for the methylation of different sequence contexts [72]. DNA cytosine methyltransferases are widely distributed among Archaeplastida but in a patchy pattern, with specific subfamily enzymes largely limited to subgroups of organisms [73]. The analysis of both phylogenesis and domain organisation of DNA cytosine methyltransferases in Archaeplastida have been conducted by Ma et al. (2017) [73] through a survey on 12 complete or near-complete algal genomes. These authors have observed that among the species analysed, only *Klebsormidium flaccidum* appears to have a set of DNA cytosine methyltransferases similar to that found in land plants, whereas homologues from other Archaeplastida algae tended to cluster independently within each subfamily. In particular, DNMT1/MET1 homologues are the methyltransferases with the widest distribution, including species belonging to Trebouxiophyceae (*Chlorella sorokiniana* and *Chlorella variabilis*) and Chlorophyceae (*C. reinhardtii* and *Volvox carteri*), as well as to the Charophyta division (*K. flaccidum*). Algal MET1 proteins share high sequence similarity in the DNA methyltransferase catalytic domain with the land plant polypeptides. Additionally, most of these algal proteins possess a D-RFD (DNA methyltransferase replication foci domain) and BAH motifs in their N-terminal extensions, as observed in the canonical enzyme. Furthermore, in *C. reinhardtii*, two additional DNMT1-related polypeptides, localised in the chloroplasts and influencing plastid DNA methylation, were found. These paralogues, lacking conserved motifs in the N-terminal region, have been termed DMT1a and DMT1b as novel DNA methyltransferase with nonselective de novo cytosine methylation activity [74,75].

According to Ma et al. (2017) [73], chromomethylase-like methyltransferases, structurally similar to land plant CMT3 except for the lack of the chromodomain in the *Chlorella* enzymes, seemed to be restricted to the chlorophytes *C. sorokiniana* and *C*. *variabilis* and to the charophyte *K*. *flaccidum*. Moreover, the homologues of DNMT3/DRM proteins, implicated in de novo DNA methylation, were found exclusively in the red alga *Cyanidioschyzon merolae* and the charophyte *K. flaccidum*. Intriguingly, the *C*. *merolae*-predicted polypeptide shows a structural organisation similar to the vertebrate DNMT3s, whereas the *K*. *flaccidum* proteins appeared more closely related to land plant DRMs. In contrast, DNMT5-related enzymes appeared limited to the Mamiellophyceae (*Bathycoccus prasinos*, *Micromonas pusilla* and *Ostreococcus lucimarinus*). The glaucophyte *Cyanophora paradoxa* contains a single DNA methyltransferase that cannot be unequivocally categorised, maybe because this alga genome has not been completely characterised and some proteins may be missing in the database [73].

Among the DNA methyltransferases encoded by microalgae, there are also other enzymes that cannot be clearly categorised. These predicted proteins contain catalytic domains somewhat related to those of the MET1 and/or the CMT subfamilies but lacking either N-terminal extensions or conserved domains in the N-terminal extensions [73]. *Chlamydomonas* DMT4 belongs to this group and it is tempting to speculate that some of these enzymes might be responsible for DNA methylation processes unique to microalgae [75].

Some species of *Chlorella* exhibit a 5-mC pattern of the nuclear genome and a complement of DNA methyltransferases (except for the lack of a DNMT3/DRM homologue) very similar to those observed in land plants. The divergence of DNA cytosine methyltransferases in other Chlorophyceae, i.e., *C*. *reinhardtii* and *V*. *carteri* from other land plants, reflect the preferential methylation of transposons and repeats in the CG, rather than in the CHG/CHH context.

Regarding Stramenopile algae, DNMT2 in *Saccharina japonica* potentially mediates the methylation in GC, CHG and CHH contexts as there is no other DNA methyltransferase encoded in the genome of this alga [76]. Instead, in the genome of diatom *Phaeodactylum tricornutum*, a peculiar set of DNMTs, as compared with other eukaryotes, was found. In this diatom, DNMT1 appears to be absent and, in addition to DNMT3, the *P. tricornutum* genome also encodes a DNMT5 protein, as well as a bacterial-like DNMT [77].

Dinoflagellates have a set of DNMTs unlike that of any other eukaryote, which is simplified in protein domain architecture but diversified in copy number [78]. *Symbiodinium* species are two orders of magnitude more abundant in copy number than observed in any other eukaryote. Moreover, well-characterised members of the DNMT1 and DNMT3 families were not found, but several paralogues of the DNMT5, DNMT6 and tRNA methyltransferase DNMT2 families were found in *Symbiodinium* genome [78].

## 3. DNA Methylation Landscape in Algae

Although, to date, DNA methylation has been studied in many plant species, including cereal crops, vegetables and trees, the role of DNA methylation in algae, especially in microalgae, is still poorly understood.

Nuclear genome 5-mC patterns have been recently profiled in several Archaeplastida microalgae, as well as in a few stramenopiles, showing low levels of DNA methylation in algae [50,72,75,76,77,79,80,81,82]. The *C*. *reinhardtii* nuclear genome is methylated at low levels (5.4, 2.6 and 2.5% in CG, CHG and CHH context, respectively) [79]; *Chlorella* sp. shows even lower CG methylation levels (4–5%) [6]. The *Picochlorum soloecismus* genome contains approximately 1.15% of cytosine methylated. Contextually, this methylation occurs in a bimodal distribution, predominately in CG sites (in ~12.1% of them) and a few in CHH and CHG sites (<1%) [81]. In *Scenedesmus acutus*, 14% of the genome is methylated and cytosine methylation occurred mainly in the symmetric CG rather than in CHG and CHH contexts (76.9% of CG, 2.6% of CHG and 1.6% of CHH context, respectively) [82]. The genome of the green alga *V. carteri* shows much lower methylation and exclusively in the CG context [50].

In the Charophytae *Klebsormidium nitens*, the global methylation patterns were comparable to that of land plants, with a preference for CG methylation and relatively high levels of CHH and CHG methylation [78].

Based on preliminary high performance liquid chromatography (HPLC) analyses of deoxycytosine methylation (5 mdC) of hydrolysed DNA from *Ectocarpus siliculosus*, DNA methylation in brown algal genomes was considered to be negligible [83]. These data indicated that the percentage of 5mdC in *E. siliculosus* is <0.035%. However, the single-base DNA methylome profiles of *S. japonica* sporophyte, female and male gametophyte revealed that ~1.4% of all cytosines are methylated in GC, CHG and CHH contexts [76]. Moreover, in *S. japonica,* approximately 57% of the methylated cytosines in the genome were methylated in a CHH context, whereas 19% and 24% are in CHG or CG contexts, respectively [76].

Until now, bisulfite sequencing data indicate a clear CG context preference in diatoms, although CHG and CHH contexts were also detected. The centric diatom *P. tricornutum* has 2.5% of cytosine methylated. In this alga, DNA methylation occurs for 1.03, 0.19 and 0.20% in CG, CHG and CHH contexts, respectively [77]. The pennate diatom *Thalassiosira pseudonata* contains 2.8% of cytosine methylated (2.57, 0.13 and 0.12% in CG, CHG and CHH sites, respectively) [72]. Instead, the *Cyclotella cryptica* genome is 61% methylated (59.4, 0.7 and 0.6% in CG, CHG and CHH sites, respectively), representing the highest amount of DNA methylation in diatom genomes observed to date [84].

The symbiotic dinoflagellates *Symbiodinium kawagutii* and *Symbiodinium minutum* exhibit a 10% difference of global methylation level; however, in both species, the highest methylation occurs at CG dinucleotides (78 and 68% of 5-mC, respectively). Moreover, the distribution of relative methylation levels at single CG sites is unimodal rather than bimodal, with most CGs showing medium-to-high methylation (mCG/CG > 0.2) [78]. By contrast, land plants have, in general, much higher levels of DNA methylation, especially at CG and CHG contexts. For example, in *A. thaliana* leaves, 30.5%, 10.0% and 3.9% methylation occurs in CG, CHG and CHH sites, respectively; rice leaves have an intermediate level of DNA methylation with 58.4% in CG, 31.0% in CHG and 5.1% in CHH sites; *Beta vulgaris* (beet) leaves show the highest levels of DNA methylation with 92.6%, 81.2% and 18.9% in CG, CHG and CHH sites, respectively [85].

As in land plants, DNA methylation can occur in the different feature regions of the algae genome, albeit differently in relation to the algal group. In some microalgae, preferential DNA methylation occurs on transposable elements, repetitive sequences and gene bodies [50,75,79,82], consistently with a role of 5-mC in the repression of transgenes, transposons and some protein-coding genes [46,50,86,87]. The *C. reinhardtii* genome exhibits one of the highest levels known in gene body methylation, with approximately 90% methylation of CG sites [6]. In this alga, DNA cytosine methylation has also been associated with the transcriptional silencing of transgenes, particularly tandem repeats [86]. Interestingly, in *Chlorella* sp. there is a large drop in methylation in the promoter region near the transcription start site, where CG methylation level is negatively correlated to gene transcription [6]. However, CHG methylation in *Chlorella* sp. is confined to repeats [6]; intriguingly, CHG and CHH methylation is also observed uniformly along chromosomes and shows little enhancement in transposons/repeats [79]. In *C. variabilis*, genes are universally CG methylated within their bodies [50]; CHG methylation is also substantial but, similarly to what occurs in land plants, is concentrated in repetitive (presumably transposon) sequences and excluded from genes [50]. In *P. soloecismus,* DNA methylation occurs globally in all genome feature regions, with greater methylation in the CG context [81]. The *V*. *carteri* genome shows preferential methylation in transposons and repeats, but a weak negative correlation between promoter methylation and transcript abundance was also observed [50]. Moreover, DNA methylation in *V*. *carteri* seems to be implicated in the transcriptional silencing of transgenes [87]. In *K. nitens*, CG methylation occurs mainly in active gene bodies and is excluded from the promoter regions. As in plants, CG gene body methylation is positively correlated with gene expression level, whereas both CHH and CHG methylation is found on silent transposable elements [78].

Intriguingly, unlike other photosynthetic eukaryotes, in the brown alga *S. japonica*, the highest methylated elements are found to be genes encoding noncoding RNAs (circular and long noncoding), whereas the methylation of TEs does not seem to play a significant role. However, even in *S. japonica* DNA methylation occurs across all the genomic feature regions [76].

In the pennate diatom *P. tricornutum*, only 3.3% of genes are methylated, whereas DNA methylation was found to be significantly high in TEs and scarce in the intergenic space, respectively. Moreover, constitutively expressed genes display low methylation levels along their transcribed sequences, whereas the differentially expressed genes have an overall higher and increasing methylation level from 5’- to 3’-end [77]. Moreover, in *C. cryptica*, the majority of highly methylated regions are repeat sequences, whereas methylation is minimal over gene sequences (3.23 and 4.14% in introns and exons, respectively) [84].

As for as chloroplast genome, *Chlamydomonas* chloroplast DNA is dynamically methylated throughout the entire life cycle: 5-mC level is low in vegetative cells; increase during gametogenesis, reaching a peak during zygote development, is probably related to the packaging and protection of chloroplast DNA in the zygospores [75].

Thus, both the distribution and function of DNA methylation in algae appear to be highly varied. In some cases, 5-mC seems to be associated with gene silencing—particularly of transposons/repeats, as in higher eukaryotes—in other DNA methylation appears to reflect algal-specific processes that still remain to be fully understood.

## 4. DNA Methylation in Algae Response to Abiotic Stress

Plants have evolved a variety of physiological and biochemical mechanisms to cope with environmental hazards. Lipid production, extreme temperatures, lighting, the amount of carbon dioxide, UV exposure, salt content and nutrient starvation are the typical abiotic stress factors that significantly affect the biochemical composition of algal cells [88,89,90,91,92,93].

Several studies describe the involvement of DNA methylation in abiotic stress response. However, the response varies for different stresses in different plant species. Most related studies have been performed on plant model organisms, especially *A. thaliana* [94] and on crops [95]. Few algal epigenetic mechanisms related to stress are known, although it has been reported that epigenetic regulation could play a positive role in stress adaptation in algae [82,96]. In this section, we provide a summary of the recent advances in the research of algal DNA methylation in response to some abiotic stress.

### 4.1. DNA Methylation and Heavy Metal Stress

Heavy metals (HMs) are significant environmental pollutants, and their toxicity is a problem of increasing significance for ecological, evolutionary, nutritional and environmental reasons [97,98]. Despite low concentrations of iron (Fe), vanadium (V), zinc (Zn) and molybdenum (Mo) are essential to carry out cellular functions of algae [99,100]; the same metals at high concentrations, also including the unessential HMs, such as arsenic (As), cadmium (Cd), chromium (Cr), lead (Pb) and mercury (Hg), cause blockages of the cell division, reduction in photosynthesis and inhibition of various thiol-group-containing enzyme activities [101,102,103].

An increasing number of studies are highlighting the role of epigenetic mechanisms in the regulation of plant HMs stress responses [104,105,106].

However, the minimal data in the literature about algal DNA methylation in response to HMs stress, concern only Cd and Cr [82,102,103,107].

Regarding Cr, one of the most diffused and toxic metals in the environment, the exposure of the green alga *S. acutus* to Cr (VI), induces specific DNA methylation changes [82]. In detail, the two *S. acutus* strains with different Cr sensitivity showed a very different methylation pattern, as revealed by whole genome bisulfite sequencing (WGBS) [82]. It was supposed that in *S. acutus,* the DNA methylation pattern might be of particular importance in reprogramming primary metabolism during prolonged Cr exposure and might define signal specificity associated with the resistance mechanism to metal stress [82,102]. Thus, under prolonged HMs exposure, algae activity is directed to enhance and/or maintain the signal levels and responses that are relevant during stress to sustain the growth through a different DNA methylation pattern as “stress memory” [82,102].

Moreover, through the immunolocalization of 5-mC, an initial lower methylation level in the Cr-tolerant strain, with respect to the wild type, was found; after Cr treatments, methylation level strongly decreased in the wild type, mainly in the heterochromatic fraction, whereas it increased in the Cr-tolerant strain [102]. Upon Cr exposure, the demethylation and the increase in euchromatin/heterochromatin ratio observed in the wild type suggest a massive gene activation in response to Cr, whereas in the tolerant strain, weaker gene activation and/or gene silencing might be involved in the response to metal exposure [102].

Cd is an unessential trace element ubiquitous in the environment [108]. After 5 days of Cd treatment, a slight increase in total 5-mC (%) level in both *C. reinhardtii* and *Scenedesmus quadricauda* was found, as revealed by global DNA methylation (5-mC) ELISA assay [103].

On the contrary, Cd exposure stimulates DNA demethylation in the red seaweed *Gracilaria dura* genome, resulting in 18.1% of hypomethylation [107]. In this alga, it was also observed that the exogenous putrescine application, either alone or in combination with selenium (Se) during Cd exposure, induced remarkable demethylation events, whereas the addition of Se and spermine (Spm), individually or in combination, significantly reduced the hypomethylation level [107]. As the induction of demethylation under Cd stress may have mediated through oxygen radicals, Se and Spm could be allied either with the elimination of Cd from enzymes that are active in metabolic processes or with the removal of ROS, stabilising the DNA methylation patterns [107].

### 4.2. DNA Methylation and Nutrient Stress

The biochemical composition of algae often responds strongly to nutrient stress. For instance, nitrogen (N), sulphur (S) and phosphorus (P) are essential macronutrients needed to promote algal growth, and they regulate metabolic activities if supplied in an acceptable form [109,110]. In algae, nutrient limitation and/or enrichment can induce significant differences in stress response and biochemical composition [1]. Nutrient stress causes the generation of free radical species in the cell and can thus result in changes in antioxidant content [111]. Several studies have investigated the influence of nutrient stress on metabolic changes in microalgae, such as the production enhancing of lipids (reviewed in [93]), carbohydrates [112,113] and/or secondary metabolites [114].

DNA methylation in plants plays a vital role in the response to nutrient changes and is involved in controlling nutrient homeostasis [115]. Although studies on nutrient deprivation in the model organism *C. reinhardtii* are primarily focused on the transcriptional programs underlying these processes [116,117,118,119], the manipulation of DNA methylation in *C. reinhardtii* cultured for many asexual generations in different environments (salt stress, phosphate starvation, and high CO_2_), affected its adaptive evolution [96].

The effects of nutrient stress, such as N, P, Fe, Zn and S stress, on DNA methylation are widely provided for plants [95]. The minimal data in the literature regarding algal DNA methylation in response to nutritional stress mainly concern nitrogen deprivation.

DNA methylation plays a role in N responses in the Chlorophyta *Picochlorum soloecismus* and potentially regulates genes that are involved in stress responses and lipid accumulation [81]. Indeed, the genome of this alga becomes hypomethylated during the growth cycle in response to N starvation. The greatest DNA hypomethylation occurs after 10 days in culture under severe N depletion conditions. During N starvation, several of the hypomethylated CG sites of *P. soloecismus* genome are annotated as genes in pathways involved in lipid biosynthesis, including diacylglycerol acyltransferase 2 (*DGAT2*) [81]. Fei et al. (2017) [28] explored the relationship between DNA methylation and transgenic silence upon N deprivation in *C. reinhardtii*. The promoter region of a diacylglycerol acyltransferase (*DGTT3*) was fused to the arylsulfatase (*ARS*) reporter gene, and it was observed that the DNA methylation rate of the transformed insertion region was high. Thus, under N deprivation, although the mRNA of the endogenous DGTT3 was significantly increased, the ARS activity of the chimeric gene expression was significantly decreased [28].

DNA methylation may also be implicated in N metabolism in diatom *P. tricornutum* [77]. The methylation profile of genes that were both methylated in normal conditions and induced in response to nitrate limitation were assessed by bisulfite sequencing in N-limiting conditions. A total of 33 genes were found to be demethylated and displayed higher expression levels than in the normal N-replete conditions [77].

According to Traller et al. (2016) [84], silicon (Si) starvation does not produce as severe phenotypes on diatoms as the nitrogen deprivation, which can cause severe cellular damage and detrimental effects. For this reason, in *P. tricornutum* DNA methylation does not control the response of Si-deplete conditions, as is reported with nitrate metabolism upon N starvation [84]. Indeed, in *C. cryptica* there is no significant change in the methylation of genes belonging to Si metabolism and/or lipid accumulation under Si-deplete and -replete conditions; nonetheless, a globally significant correlation of methylation is observed between the two growing conditions in this alga [84].

Regarding S deprivation stress, in the Cr-tolerant strain of *S. acutus* it was observed that epigenetic mechanisms play an important role in the modulation of the sulphate pathway [82]. The hypomethylation of the *SaSULTR1* promoter (encoding for a H^+^/SO_4_^2−^ transporter) is linked to its overexpression in this strain [82] and S starvation induced strong *SaSULTR1* expression [120]. Thus, Ferrari et al. [120] hypothesised that DNA methylation is also involved in response to S availability in *S. acutus*.

### 4.3. DNA Methylation and Temperature Stress

Fluctuations in temperature affect numerous metabolic activities in algae. For instance, high temperature stimulates the production of active oxygen species that damage photosynthetic machinery due to the suppression of activities of antioxidant enzymes [121]. Heat stress can also induce changes in the composition and production of lipids [122], as well as many other macromolecules of algae [123]. Low temperature also shows a decrease in growth due to photo-oxidative damage of several macromolecules [124].

Increasing evidence has indicated that DNA methylation plays important roles in the response to temperature stress in land plants [28,125]. Nevertheless, minimal data for algae are reported, which only regard some rhodophytes and symbiotic dinoflagellates [78,126].

By using the MSAP (methylation-sensitive amplified polymorphism) technique, it was observed that both high and low temperature stress induced some changes in the methylation pattern of the red alga *Gracilariopsis lemaneiformis* [126]. Furthermore, the high temperature could induce more cytosine methylation/demethylation events than low-temperature treatment [126]. The increase in DNA methylation level under temperature stress may inhibit the expression of some genes contributing to the temperature adaptation in *G. lemaneiformis* [126].

In the thermoacidophile red alga *G. sulphuraria*, after continuous cold stress (14 °C below its optimal growth temperature) for more than 100 generations, CpG islands located in the intergenic region accumulated a significant number of variants, which is likely a sign of epigenetic remodelling [127]. Moreover, the cold-adapted samples grew ∼30% faster than the starting population. The significant growth enhancement of *G. sulphuraria* grown at low temperatures is driven mainly by mutations in genes involved in the cell cycle, gene regulation and signal transfer, as well as mutations that occurred in the intergenic regions, possibly changing the epigenetic methylation pattern and altering the binding specificity to *cis*-regulatory elements [127]. 

WGBS was performed in the dinoflagellates *S. kawagutii* and *S. minutum* at different temperature conditions, and global cytosine methylation levels provided similar results across samples of the same species, indicating the minimal effect of thermal stress on global methylation levels [78]. However, through a transcriptomic approach, it was observed that in the dinoflagellate *Biecheleriopsis adriatica* under cold stress, the “methylation” category had the highest difference between up- and down-regulated genes in gene ontology analysis, suggesting that cold stress may affect DNA methylation and gene expression in this microalga [128].

## 5. Conclusions

In this review, we briefly discussed DNA methylation and its possible role in abiotic stress responses in algae. Today, the literature data suggest that methylome dynamics under stressful conditions depend on both algal species and the kind of abiotic stress. Although increasing studies on DNA methylation are being performed in plants, this review highlights the scarcity of data in this field for algae. More studies would be helpful in deciphering algae methylation in order to unfold its epigenetics role in stress response, as well as in the network of the different biological activities of these organisms. A better comprehension of epigenetic responses upon abiotic stress in algae could be useful to sustain algal growth and development under variable environmental conditions. From a practical perspective, these findings might enable the engineering of algae to be more resilient to stress. Moreover, by modifying with epigenetics, the expression of specific genes, which significantly affect the biochemical processes, might tune the cellular and physiological homeostasis to produce useful metabolites of interest.

## Data Availability

Not applicable.
